# Retraction: Effects of curcumin on glycemic control and lipid profile in prediabetes and type 2 diabetes mellitus: A systematic review and meta-analysis

**DOI:** 10.1371/journal.pone.0233919

**Published:** 2020-05-22

**Authors:** The PLOS ONE Editors

After publication of this article [1], concerns were raised about aspects of the methods applied and whether the included studies adhered to the reported inclusion and exclusion criteria. *PLOS ONE* conducted a post-publication review of the article, involving senior members of the Editorial team, a member of the Editorial Board, and a statistical reviewer with expertise in systematic reviews and meta-analyses. Based on the outcome of this assessment and follow-up discussions with the authors, we have concerns about the reliability of the results reported in this article and whether the conclusions are adequately supported.

Specific concerns include:

- The study is reliant on studies that are highly heterogeneous. The authors conducted a sensitivity analysis, but the statistical reviewer advised that the large degree of heterogeneity would need to be explored further and cannot be addressed in a random effects meta-analysis.
- Concerns were raised about the quality of included studies, which have relatively low sample sizes and were found by the statistical reviewer to be poorly reported. Several of the included studies lack information about key aspects of trial design such as blinding, concealment, and issues around missing data. Also, the timepoint for analysis is not clear or consistent across included studies. Some of these issues were noted in the article’s Results and/or Discussion.
- The meta-analysis does not address issues such as losses to follow-up, missing data, imbalances at baseline, and regression to the mean effects. The corresponding author commented that results were calculated based on 118 patients (of 120 at baseline) in the intervention group and 116 patients (of 117 at baseline) in the control group, and as such they considered that loss to follow-up would not substantially impact the study’s validity.
- One cannot determine whether ascertainment of trials for the meta-analysis was correct and complete based on the information reported in the article. The Materials and methods do not clearly report reasons for exclusion, and the reasons for excluding 359 articles were not provided.
- Nine trials were excluded because they tested “curcumin as combination”. This was not mentioned as an exclusion criterion in the Materials and methods section, and in at least nine of the included trials participants used other medications in addition to the curcumin treatment. The authors clarified that the term ‘combination’, applied in the inclusion criteria, referred to whether the curcumin preparation included other extracts. It did not refer to the background therapy of participants or other medications administered during the trial. The authors noted that they did not have information about participants’ comorbidities or other ongoing treatments which may impact HbA1c and fasting plasma glucose. This issue was noted in the article’s Discussion.
- The statistical reviewer advised that the summary of the results dichotomizes significance and does not provide information about the degree of uncertainty. They also advised that last observation carried forward (LOCF) imputation of missing outcome data would reward early dropout and favor curcumin in this study.
- The statistical reviewer advised that given the number and size of trials included, it is difficult to assess publication bias as would be needed to clarify whether the article’s conclusions are supported by the available evidence. The study included analyses of three studies evaluating glycemic outcomes in participants with prediabetes, and eight trials (6 with HbA1c outcome data, 8 with fasting blood glucose data) assessing glycemic outcomes in type 2 diabetes mellitus. Funnel plot and Egger’s test were used to test publication bias in cases where there were five or more studies. The power of such tests to detect publication bias is dependent on the number of included studies, and is limited in cases where there is a small number of included studies; per the Cochrane Handbook, funnel plots should not be used in cases where there are fewer than 10 studies [2–4].
- Overall, the conclusions overstate what can be drawn from the results of the meta-analysis, given the limitations of the study and the analyses which were reported.

Although some of the above issues could be addressed through improved reporting and further analysis, the article’s conclusions are not supported by the available literature in light of the above concerns. Therefore, the *PLOS ONE* Editors retract this article. We regret that these issues were not fully addressed during the article’s pre-publication peer review.

The authors (NP, NS, PDMK, KD) did not agree with retraction and stand by the article’s findings.
